# Adunctin E from *Conamomum rubidum* Induces Apoptosis in Lung Cancer via HSP90AA1 Modulation: A Network Pharmacology and In Vitro Study

**DOI:** 10.3390/ijms252111368

**Published:** 2024-10-22

**Authors:** Iksen Iksen, Natsaranyatron Singharajkomron, Hien Minh Nguyen, Hanh Nhu Thi Hoang, Duc Viet Ho, Varisa Pongrakhananon

**Affiliations:** 1 Department of Pharmacology and Physiology, Faculty of Pharmaceutical Sciences, Chulalongkorn University, Bangkok 10330, Thailand; ikseniksen08@gmail.com (I.I.); natsaranyatron.s@gmail.com (N.S.); 2 Faculty of Pharmacy, Ton Duc Thang University, Ho Chi Minh City 700000, Vietnam; ngyenminhhien@tdtu.edu.vn; 3 Faculty of Engineering and Food Technology, Hue University of Agriculture and Forestry, Hue University, Hue City 49000, Vietnam; htnhanh@hueuni.edu.vn; 4 Faculty of Pharmacy, Hue University of Medicine and Pharmacy, Hue University, Hue City 49000, Vietnam; hvietduc@hueuni.edu.vn; 5 Preclinical Toxicity and Efficacy Assessment of Medicines and Chemicals Research Unit, Chulalongkorn University, Bangkok 10330, Thailand

**Keywords:** adunctin E, apoptosis, *Conamomum rubidum*, lung cancer, network pharmacology, molecular docking

## Abstract

Lung cancer stands out as a leading cause of death among various cancer types, highlighting the urgent need for effective anticancer drugs and the discovery of new compounds with potent therapeutic properties. Natural sources, such as the *Conamomum* genus, offer various bioactive compounds. Adunctin E (AE), a dihydrochalcone derived from *Conamomum rubidum*, exhibited several pharmacological activities, and its potential as an anticancer agent remains largely unexplored. Thus, this study aimed to elucidate its apoptotic-inducing effect and identify its molecular targets. The network pharmacology analysis led to the identification of 71 potential targets of AE against lung cancer. Subsequent gene ontology (GO), Kyoto Encyclopedia of Genes and Genomes (KEGG), and Reactome pathway enrichment analyses revealed the involvement of these targets in cancer-associated signaling pathways. Notably, HSP90AA1, MAPK1, and PIK3CA emerged as key players in apoptosis. In silico molecular docking and dynamic simulations suggested a strong and stable interaction between AE and HSP90AA1. In vitro experiments further confirmed a significant apoptotic-inducing effect of AE on lung cancer cell lines A549 and H460. Furthermore, immunoblot analysis exhibited a substantial decrease in HSP90AA1 levels in response to AE treatment. These findings support the potential anticancer activity of AE through the HSP90AA1 mechanism, underscoring its promise as a novel compound worthy of further research and development for anti-lung cancer therapy.

## 1. Introduction

Lung cancer remains a significant global health concern, with high incidence and mortality rates [1]. It is primarily categorized into two main subtypes: non-small cell lung cancer (NSCLC), which constitutes approximately 85% of cases, and small-cell lung cancer (SCLC), which accounts for the remaining 15% [2,3]. Unfortunately, nonspecific lung cancer-associated symptoms often lead to its late diagnosis or diagnosis at an advanced stage [4]. Current standard therapies for lung cancer include surgery, radiotherapy, chemotherapy, and targeted therapy [5]. Despite advances in lung cancer therapy, therapeutic resistance, cancer recurrence, and metastasis often develop after multiple treatments, and the mortality rate is gradually increasing [6,7,8], highlighting the urgent need for new anticancer drugs.

Plants are promising sources of natural biological actives for drug development. Compounds, particularly those extracted from the genus *Conamomum*, a synonym of the genus *Amomum*, in the Zingiberaceae family, have demonstrated pharmacological activities such as antimicrobial and antioxidant activities and have long been used in traditional medicine for their anti-inflammatory and fever-reducing properties [9,10,11,12]. A recent study has reported the potent cytotoxicity of compounds such as adunctin E (AE), a dihydrochalcone isolated from *Conamomum rubidum* in lung cancers [13]. However, the anticancer activity of AE and its underlying molecular mechanism remain unexplored.

Recently, bioinformatics has significantly advanced in drug research and development by not only identifying potential therapeutic targets but also aiding in drug design [14,15]. Network pharmacology approaches have emerged as invaluable tools in this process and facilitated the identification of potential molecular targets of novel compounds promptly and precisely [16,17]. These approaches, through integration with multiple bioinformatic databases, provide insights into the molecular targets of diseases and predict targets of new compounds. Furthermore, the mechanism of action was investigated through the pathway analysis. In silico molecular docking allows for the prediction of interactions between new lead compounds and identified target molecules [18,19]. Importantly, targets can be validated using in vitro experimental models, providing essential preclinical data of new lead compounds for further investigation and ultimately expediting the drug discovery [20].

In this study, we aimed to identify the molecular targets of AE by integrating network pharmacology methodology, investigate its interaction with molecular targets using in silico molecular docking, and validate these molecular targets through in vitro lung cancer cell-based experiments. The findings of this study could offer insights into the anticancer activity of AE and elucidate its molecular mechanisms of action for potential therapeutic development.

## 2. Results

### 2.1. Pharmacokinetic Parameters and Target Identification of AE in NSCLC1

**Figure 1:**
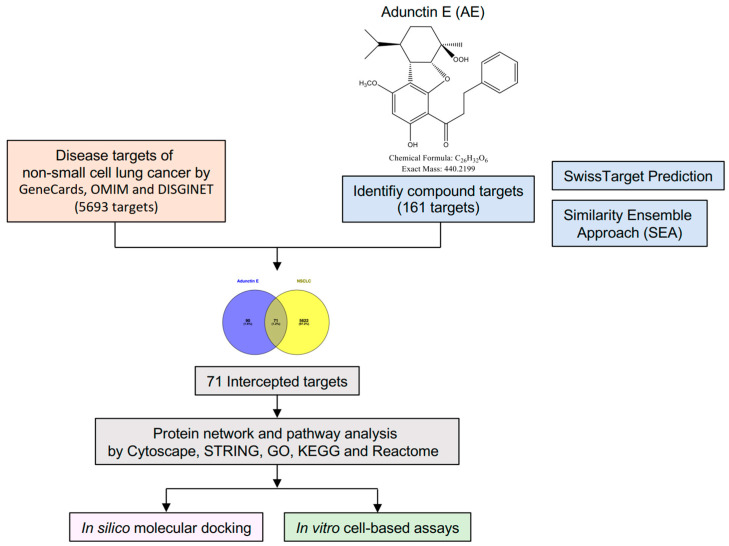
Workflow for the investigation of the molecular targets of adunctin E (AE).

**Figure 2:**
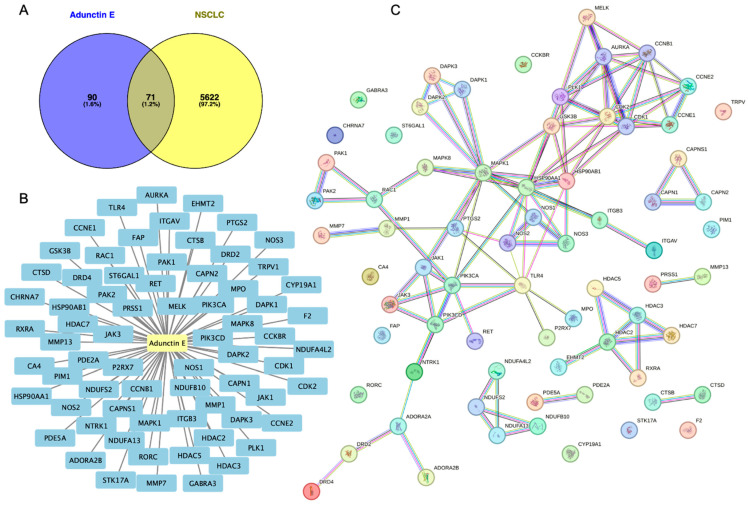
Molecular target identification of adunctin E. (**A**) This Venn diagram represents targets of adunctin E (blue), targets in non-small cell lung cancer (NSCLC, yellow), and common targets between the compound and the disease. (**B**) A compound–target network was constructed by Cytoscape 3.9. Active components were labeled in yellow, whereas the 71 common targets were highlighted in blue. (**C**) The protein–protein interaction (PPI) network of the common targets was analyzed by importing 71 common targets to the search tool from the STRING.

The workflow is illustrated in Figure 1. Pharmacokinetic parameters were analyzed by pkCSM, indicating that AE has high intestinal absorption, low blood–brain barrier permeability, and less toxic (Table S1). Additionally, AE was predicted to inhibit CYP enzymes, which may influence both its therapeutic efficacy and safety profile. Molecular targets of AE were retrieved from the Swiss Target Prediction database and SEA, extracting 160 predicted targets (Table S2). NSCLC-associated targets were obtained from GeneCards, OMIM, and DisGeNET, yielding a total of 5693 targets after removing duplicates. A Venn diagram identified 71 common targets of AE and NSCLC (Figure 2A and Table S3). Subsequently, a compound–target interaction network was constructed using Cytoscape 3.9.1 (Figure 2B). In the network, active components were labeled in yellow, whereas the 71 common targets were highlighted in blue.

### 2.2. Construction of PPI Network and Enrichment Analyses of GO, KEGG and Reactome Pathways

**Figure 3:**
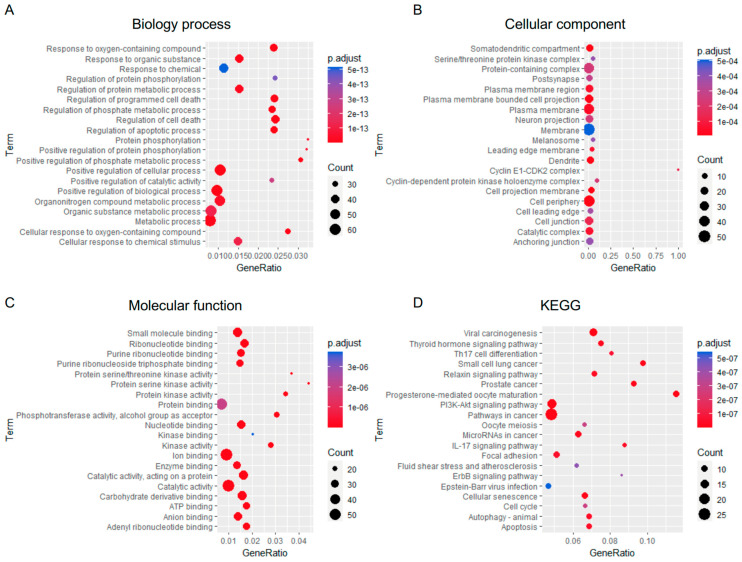
Gene ontology (GO) and Kyoto Encyclopedia of Genes and Genomes (KEGG) pathway enrichment analyses were conducted on the potential targets of adunctin E in non-small cell lung cancer (NSCLC). Data analyzed in STRING were imported to RStudio with the ggplot2 package. The GO terms examined include (**A**) biological process, (**B**) cellular component, and (**C**) molecular function. (**D**) The KEGG pathway associated with these common targets was analyzed.

To evaluate potential associations among targets, the PPI network of the 71 common targets was analyzed using the STRING (Figure 2C). In the network, nodes represented common targets, and edges indicated an association between nodes, including neighboring, fusion, and co-occurring genes. Sixty-four common targets were connected, whereas seven nodes did not show interactions. Subsequently, the common targets were subjected to GO functional annotation analyses which were performed by mapping the target genes to Gene Ontology categories and conducting enrichment analysis to identify significantly enriched terms. The top 20 enriched biological processes associated with cancer included responses to chemical, metabolic process regulation, regulation of cell death and apoptotic process, and protein phosphorylation, according to the degree of significance (Figure 3A). In cellular component associations with lung cancer, the targets were mainly found in the cell projection membrane, cell periphery, plasma membrane, catalytic complex, and cyclin E1-cyclin-dependent kinase 2 (CDK2) complex (Figure 3B). For molecular functions, the targets were involved in ribonucleotide binding, protein kinase activity, catalytic activity, carbohydrate derivative binding, and ion binding (Figure 3C).

In the KEGG pathway analysis, these targets participated in cancer-related pathways such as viral carcinogenesis, the PI3K–AKT signaling pathway, microRNAs in cancer, cellular senescence, cell cycle, autophagy, and apoptosis (Figure 3D). Furthermore, the Reactome pathway analysis identified several significantly enriched pathways, highlighting key biological processes related to the input gene set. The analysis used p-adjust values to ensure statistical significance. The data suggested that the molecular targets of AE in NSCLC were associated with signal transduction, receptor tyrosine kinase signaling, extracellular matrix degradation, regulation of transcription of cell cycle genes by p53, MAPK family signaling cascades, apoptosis, cell cycle, and PI3K/AKT signaling in cancer (Table 1).

### 2.3. Potential Target Identification

**Figure 4:**
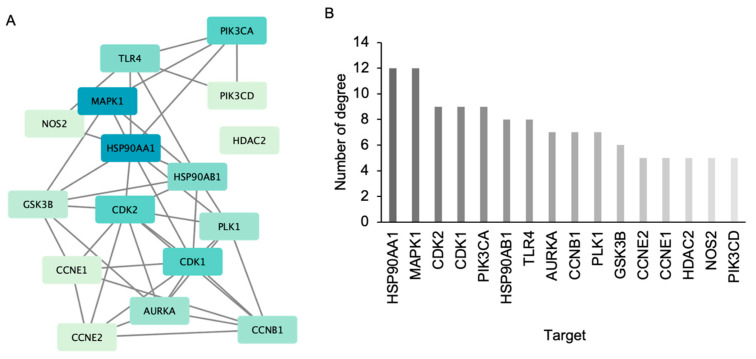
(**A**) The association of common molecular targets of adunctin E and non-small cell lung cancer was constructed by Cytoscape 3.9.1. The top 16 common targets with the highest degree scores were generated using the cytoHubba plugin. Colors ranging from dark blue to light green indicate a higher to lower score of degree. (**B**) Plot of the degree values of the top 16 common targets.

The common target network obtained from STRING was imported into Cytoscape (Figure 4A), and the topology analysis highlighted the top 16 molecules based on their degree of connectivity (Figure 4B and Table S4). These molecules, appearing dark to light green according to their degree scores, are potential targets of AE in NSCLC. Among the identified targets, the key regulators implicated in NSCLC pathogenesis included HSP90AA1, MAPK1, CDK2, cyclin-dependent kinase 1 (CDK1), phosphatidylinositol-4,5-bisphosphate 3-kinase catalytic subunit alpha (PIK3CA), heat shock protein 90 alpha family class B member 1 (HSP90AB1), toll-like receptor 4 (TLR4), aurora kinase A (AURKA), cyclin B1 (CCNB1), polo-like kinase 1 (PLK1), glycogen synthase kinase 3 beta (GSK3B), cyclin E2 (CCNE2), cyclin E1 (CCNE1), histone deacetylase 2 (HDAC2), nitric oxide synthase 2 (NOS2), and phosphatidylinositol-4,5-bisphosphate 3-kinase catalytic subunit delta (PIK3CD).

Because of the crucial role of apoptosis induction in anticancer therapy, we focused primarily on potential targets classified as apoptosis regulators. Among these targets, HSP90AA1, MAPK1, and PIK3CA emerged as promising candidates based on their degree scores in the network analysis.

### 2.4. Molecular Docking and Molecular Dynamic Analysis of AE Target Interactions

**Figure 5:**
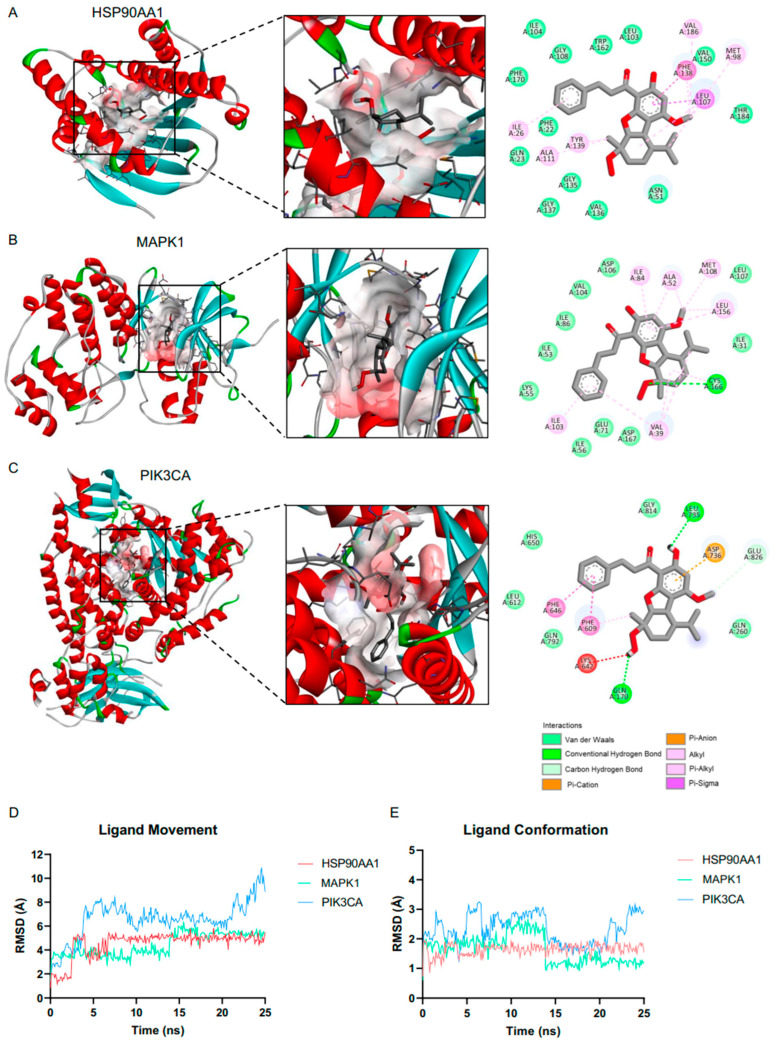
Molecular docking and molecular dynamics between adunctin E and protein targets: 2D and 3D interactions between adunctin E and HSP90AA1 (**A**), MAPK1 (**B**), and PIK3CA (**C**). Root mean square deviation for ligand movement (**D**) and ligand conformation (**E**). Simulation between 22 adunctin E and HSP90AA1 (red), MAPK1 (green), and PIK3CA (blue) for 25 ns were plotted.

Molecular docking experiments were performed to analyzed potential interactions between AE and the identified apoptosis regulators. The results revealed that AE binds to these targets through a combination of hydrogen bonding, van der Waals forces, hydrophobic interaction, and electrostatic interactions (Figure 5A–C). Specifically, the binding energies of AE with HSP90AA1, MAPK1, and PIK3CA were −10.1, −7.7 and −8.1 kcal/mol, respectively (Table 2). The ligand efficiency between AE and HSP90AA1 was notably high, indicating significant contributions from each heavy atom in the ligand to the binding interaction with the target protein. Conversely, the interactions between AE and either MAPK1 or PIK3CA exhibited moderate ligand efficiency.

Furthermore, molecular dynamics simulations were performed to investigate the stability and conformational changes of the ligand–target protein complexes over time (Figure 5D,E). The ligand movements for AE with HSP90AA1, MAPK1, and PIK3CA were 4.53 ± 0.07 Å, 4.44 ± 0.06 Å, and 6.58 ± 0.1 Å. The conformational changes in the ligands were 1.64 ± 0.01 Å, 1.64 ± 0.03 Å, and 2.33 ± 0.03 Å for HSP90AA1, MAPK1, and PIK3CA, respectively. Notably, the RMSD of the ligand’s movement and conformation with HSP90AA1 remained relatively stable up to the 25 ns mark. In contrast, greater fluctuations were observed in the interactions between AE and both MAPK1 and PIK3CA. These findings suggest that AE forms particularly stable complexes with HSP90AAA1, indicating the strongest interaction among the tested proteins. This highlights the potential of AE as a therapeutic agent in NSCLC by targeting key signaling pathways involved in apoptosis regulation.

### 2.5. In Vitro Apoptosis-Inducing Effect of AE on Nsclc

**Figure 6:**
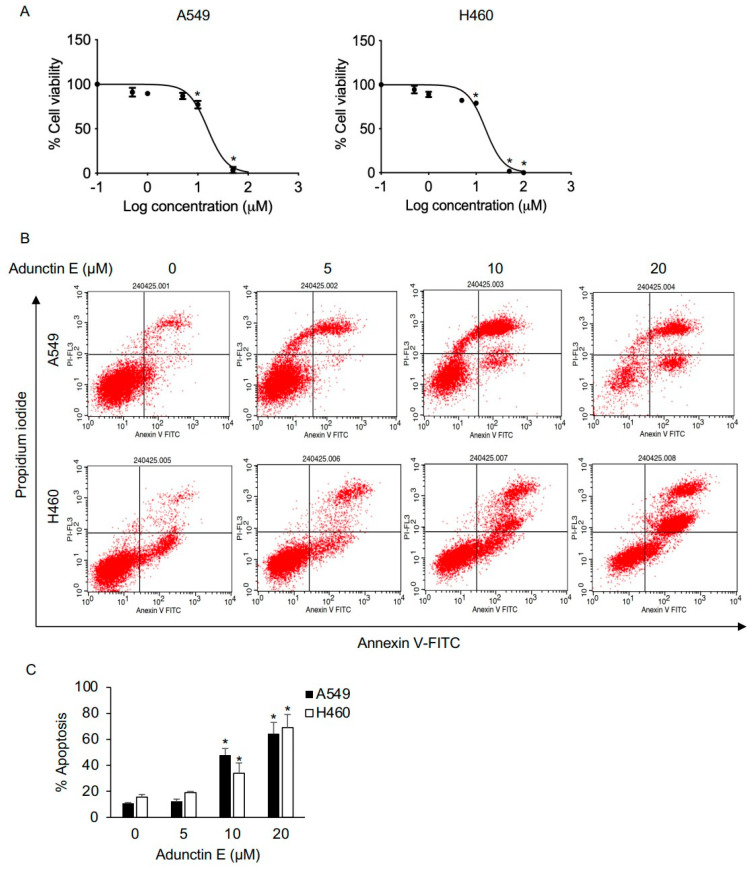
In vitro cytotoxicity and apoptosis induction of adunctin E. (**A**) A549 and H460 cells were treated with adunctin E (0–100 μM) for 48 h. Cell viability was determined by MTT assay. Plots are presented as a percentage of cell viability. (**B**) Apoptosis cells were evaluated by annexin-V/propidium iodide (PI) staining. Representative histograms from the flow cytometry analysis are shown. (**C**) The number of early (annexin-V^+^, PI^–^) and late (annexin-V^+^, PI^+^) apoptotic cells were plotted. Data are presented as the mean ± SEM (n = 3). * *p* < 0.05 vs. untreated control cells.

To assess the in vitro cytotoxic effects of AE on NSCLC, A549 and H460 cells were treated with varying concentrations of AE (0–100 μM) for 48 h, and a cytotoxic assay using MTT was performed. The results demonstrated a significant reduction in the viability of NSCLC, with an IC_50_ of 15.72 ± 3.37 and 15.71 ± 3.43 μM in A549 and H460 cells, respectively (Figure 6A). Furthermore, AE-induced cell apoptosis was evaluated by treating cells with similar conditions, followed by analysis with annexin-V/propidium iodide (PI) staining. The result revealed that the number of early (annexin-V^+^, PI^–^) and late (annexin-V^+^, PI^+^) apoptotic cells gradually increased in a dose-dependent manner, with 48% and 64% observed in A549 cells and 34% and 68% in H460 cells treated with 10 and 20 μM of AE, respectively (Figure 6B,C).

### 2.6. AE Downregulates HSP90AA1 Expression

**Figure 7:**
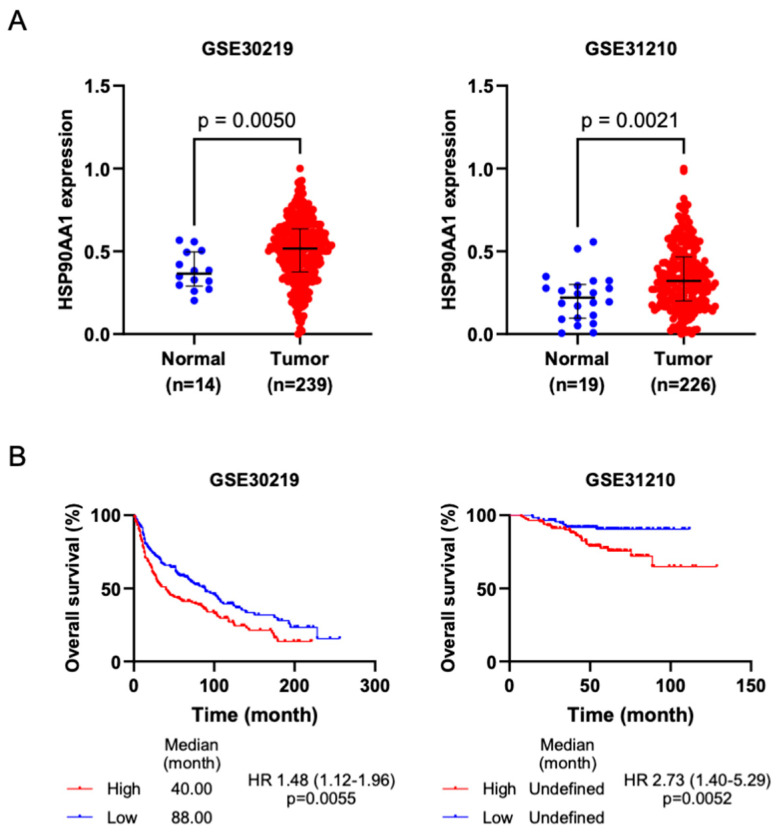
HSP90AA1 is a molecular target of adunctin E. (**A**) HSP90AA1 expression was upregulated in lung cancer. HSP90AA1 expressions in both normal lung (blue circles) and lung tumor (red circles) tissues were assessed utilizing GEO data. (**B**) Kaplan–Meier survival analysis of lung cancer patients who had high and low HSP90AA1 expressions from the GEO cohort. HR, hazard ratio.

Based on the above results, HSP90AA1 emerged as a top potential candidate targeted by AE in NSCLC. To underscore the clinical relevance of HSP90AA1 in lung cancer, an analysis of its differential expression and overall survival was performed. Data revealed a significant upregulation of HSP90AA1 mRNA expression in lung cancer tissues compared with lung normal tissues in all datasets (Figure 7A). Furthermore, lung cancer patients with high HSP90AA1 levels exhibited lower overall survival than those with low HSP90AA1 expression (Figure 7B). These finding suggests the prognostic significance of HSP90AA1 in lung cancer, highlighting it as a potential therapeutic target.

**Figure 8:**
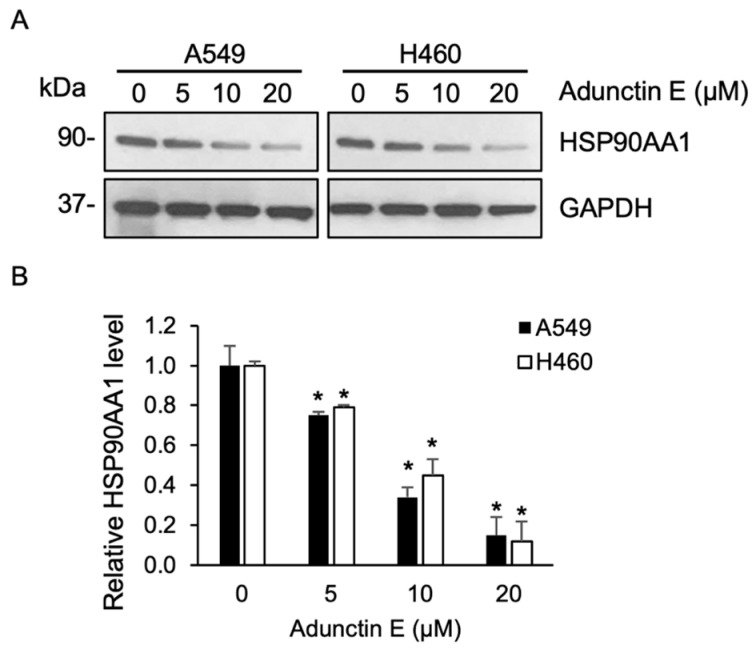
(**A**) A549 and H460 cells were treated with adunctin E (0–20 μM) for 48 h. The expression of HSP90AA1 was analyzed by immunoblotting. Blots were reprobed with anti-GAPDH antibody to ensure equal loading. Representative blots from triplicate independent experiments are shown. (**B**) HSP90AA1 protein levels were quantified and normalized with those of GAPDH. Relative HSP90AA1 protein levels were plotted. Data are presented as mean ± SEM (n = 3). * *p* < 0.05 vs. untreated control cells.

Further, to investigate the effect of AE on HSP90AA1 levels, cells were treated with various concentrations of AE. Immunoblot analysis demonstrated a gradual decrease in HSP90AA1 levels in a dose-dependent manner (Figure 8). At a concentration of 20 μM, AE substantially reduced HSP90AA1 expression levels to 0.15- and 0.12-fold in A549 and H460 cells, respectively, compared with the untreated control cells. This finding confirms that HSP90AA1 serves as a molecular target of AE in facilitating lung cancer cell apoptosis.

## 3. Discussion

The landscape of anticancer drug discovery and research, particularly in the context of lung cancer, presents ongoing challenges. Despite advances in current therapeutic interventions, the OS rates have gradually increased [1]. Our findings shed light on the remarkable anticancer properties of AE against lung cancer. Using the network pharmacology approach, HSP90AA1 was identified as a significant molecular target of AE. In silico assays revealed a potent and stable interaction between AE and HSP90AA1. Furthermore, AE induced apoptosis of lung cancer cells through this mechanism. This underscores AE as a promising candidate for further anticancer drug research and development.

Network pharmacology offers various advantages in drug research and discovery. This high-throughput approach has significantly accelerated the drug discovery process while minimizing costs [21,22]. Enabling the identification of potential molecular targets of various biologically active compounds has garnered significant interest in the field of drug discovery [16]. In this study, we identified 71 possible AE targets in lung cancer. Pathway analysis using GO, KEGG, and Reactome further narrowed down potential molecular targets, showing notable associations with apoptosis signaling pathways in cancer, particularly for HSP90AA1, MAPK1, and PIK3CA.

Apoptosis dysregulation is a recognized hallmark of cancer [23]. Apoptosis, or programmed cell death, is crucially involved in normal physiologies, including embryonic development and tissue homeostasis, without mediating inflammatory responses or being harmful to neighboring cells [24,25]. An abnormal apoptosis mechanism contributes to the pathogenesis of various diseases including cancers [26]. Cancer cells often acquire deregulated apoptotic signaling either by the upregulation of antiapoptotic and/or prosurvival proteins or the downregulation of proapoptotic signaling [27,28,29]. Therefore, cancer therapeutics target the editing of these apoptotic signaling pathways. This study also demonstrated the potent cytotoxic effect of AE on lung cancer cells, mediated through apoptosis mechanisms.

Notably, HSP90AA1, MAPK1, and PIK3CA emerged as potential molecular targets for AE in inducing apoptosis. Notably, in silico experiments revealed that AE formed the strongest and most stable interaction with HSP90AA1, suggesting that this protein may play a critical role in the mediating of apoptotic effects by AE. Despite strong support from molecular docking and dynamic simulation studies, there is still a possibility of indirect effects. Additional experimental validation is required to confirm the specificity and mechanism of this interaction. Moreover, it is plausible that AE exerts its effects through multiple pathways, and indirect modulation of other proteins cannot be excluded. HSP90AA1, a member of the molecular chaperone network, is upregulated in response to cellular stress [30] and has been implicated in cancer aggressiveness and poor survival outcomes in various cancers [31,32,33]. It interacts with key signaling targets such as MAPK and AKT that regulate the stability and functions of the targets, contributing to cancer aggressiveness including apoptosis resistance and metastasis [34,35,36,37]. An inhibitor of HSP90AA1 was reported to remarkably induce apoptosis and suppress cell proliferation in lung cancer through an ERK/AKT-dependent mechanism [34] and to induce autophagic cell death in osteosarcoma by suppressing AKT/mTOR signaling [37], highlighting its importance in cancer biology.

In this study, we demonstrated that AE downregulates HSP90AA1, a critical protein involved in cancer cell survival. Our findings indicate that AE binds to the N-terminal domain of HSP90AA1, specifically interacting with THR184 (Figure S1). This interaction is likely to disrupt the ATP-binding activity of HSP90AA1, which is essential for its chaperone function. By interfering with the N-terminal ATPase activity, AE may induce structural instability in HSP90AA1, impairing its proper folding and function. Consequently, the misfolded HSP90AA1 is likely targeted for degradation through the proteasome pathway, which plays a significant role in the clearance of misfolded proteins [38]. While our current study primarily focuses on protein-level effects, we acknowledge the need for further mechanistic investigations, such as RNA-based assays, to fully elucidate how AE modulates protein levels specifically, whether this modulation occurs via the inhibition of translation or through targeted degradation.

MAPK1 (also known as ERK2) is a crucial target of HSP90AA1, whereas PIK3CA serves as an upstream kinase for AKT signaling [37,39]. This suggests that HSP90AA1 inhibition holds significant promise as a therapeutic approach for cancers, given its central role in regulating key signaling pathways involved in cancer progression. However, it is important to acknowledge that HSP90AA1 operates within broader, interconnected signaling networks that include the MAPK and PI3K/AKT pathways [34,37]. These proteins are frequently co-regulated in various cancer contexts, and their overlapping roles may limit the specificity of targeting HSP90AA1 alone. Future investigations will focus on elucidating the effects of AE on the MAPK, PI3K, and AKT pathways, thereby enhancing the therapeutic potential of AE by demonstrating its influence on multiple cancer-related mechanisms. This multi-target approach could provide a more comprehensive strategy for addressing the complex signaling networks in lung cancer. In addition, further studies, particularly in animal models, are warranted to validate the anticancer efficacy of AE and elucidate its mechanism of action.

## 4. Materials and Methods

### 4.1. Chemicals and Reagents

3-(4,5-dimethylthiazol-2-yl)-2,5-diphenyltetrazolium bromide (MTT) was purchased from Sigma-Aldrich (St. Louis, MO, USA). AE (Figure 1) was isolated from *Conamomum rubidum*, and its 1H and 13C nuclear magnetic resonance (NMR) spectra were reported previously [13]. Briefly, the isolation of AE from *Conamomum rubidum* involved extracting with methanol and partitioning with n-hexane. In addition, the n-hexane fraction was separated using silica gel chromatography by using an n-hexane-acetone solvent system. The pharmacokinetic parameters were analyzed by pkCSM [40].

### 4.2. Identification of the Targets of AE and Nsclc-Related Genes

The possible targets of AE were retrieved from the Swiss Target Prediction database [41] and the Similarity ensemble approach (SEA) [42]. Key NSCLC-associated molecular targets were obtained from GeneCards [43], Online Mendelian Inheritance in Man (OMIM) [44], and DisGeNET [45]. The compound–target network was visualized using Cytoscape version 3.9.1 [46]. The common targets of AE and NSCLC were identified by Venny version 2.1.0 [47] and presented in a Venn diagram.

### 4.3. Construction of the Protein–Protein Interaction Network

The protein–protein interaction network was constructed using STRING version 11.5 [48] by incorporating the common targets of AE and NSCLC. The protein type was specified as “*Homo sapiens*” with a confidence level set to 0.7, and other parameters were set to default values. To create the interaction network, protein interaction relationships were imported into Cytoscape version 3.9.1. Subsequently, top targets were analyzed using the cytoHubba plugin [49]. Core proteins with the highest degree values were subjected to further analyses.

### 4.4. Bioinformatic Analyses of Gene Ontology (Go), and Kyoto Encyclopedia of Genes and Genomes (Kegg), and Reactome Pathways

GO and KEGG were retrieved from the STRING version 11.5 database by importing the common targets of AE and NSCLC. GO analysis was performed to explore the functionality of genes, including biological processes, cellular components, and molecular functions [50]. In NSCLC, the putative molecular mechanisms of AE were elucidated through KEGG pathway enrichment analyses [51]. Data were visualized by R software version 1.4.1717 with ggplot2 [52]. Results were visualized as a bubble plot, with the *X*-axis representing the gene ratio, the *Y*-axis denoting the GO terms, and bubble size and color indicating the number of associated genes and their statistical significance.

Reactome pathway analysis was also employed to investigate pathways associated with the identified targets [53]. Targets were mapped to corresponding pathways in the Reactome database. A statistical method was applied to assess pathway enrichment, using an adjusted *p*-value threshold (e.g., <0.05) to determine significance. The enriched pathways were then visualized and interpreted to understand potential alterations in biological processes and signaling pathways relevant to the study.

### 4.5. Molecular Docking and Dynamics

The X-ray crystal structures of the potential targets were retrieved from the Protein Data Bank (PDB). The structure of AE was drawn by ChemDraw Ultra version 15.0 (Perkin Elmer, Waltham, MA, USA). Molecular docking studies of AE with the protein targets were conducted using the PyRx Virtual Screening Tool version 0.8. Ligand conformations that exhibited the highest clusters were analyzed to determine their free binding energies (ΔG). The binding interactions between the ligands and target proteins were evaluated using PyMOL version 2.4 (Schrödinger, Portland, OR, USA) and BIOVIA Discovery Studio Visualizer 2022 (Biovia, San Diego, CA, USA). Molecular dynamics simulations were performed using Yasara software (https://www.yasara.org/) with the AMBER14 force field. The simulations were conducted at a temperature of 298 K and pH of 7.4, lasting for 25 ns. The default macro md_run.mcr and md_analyse.mcr. were employed for analyses. The root mean square deviation (RMSD) graphic was generated using RStudio software version 1.4.1717 [54].

### 4.6. Gene Expression Datasets and Differential Expression Analysis

mRNA expression data were obtained from the Gene Expression Omnibus (GEO) database [54]. Two GEO datasets, namely, GSE30219 (tumors, n = 239; normal lung tissues, n = 14) and GSE31210 (tumors, n = 226; normal lung tissues, n = 19), were analyzed. The expression levels of HSP90AA1 in normal and tumor lung tissues were compared.

### 4.7. Survival Analysis

mRNA expression data of HSP90AA1 and lung cancer survival information were obtained from the GEO databases (GSE30219, n = 239; GSE31210, n = 226) [55]. According to the median expression level, patients were categorized into high and low HSP90AA1 expression groups. The overall survival rates in these two groups were compared using Kaplan–Meier plots generated by Prism 10 version 10.2.3 (GraphPad Software, Boston, MA, USA). A log-rank *p*-value < 0.05 was considered statistically significant.

### 4.8. Cell Culture

Human NSCLC H460 and A549 cells were purchased from the American Type Culture Collection (ATCC, Manassas, VA, USA). H460 were cultured in Roswell Park Memorial Institute (RPMI) Medium, while A549 cells were maintained in Dulbecco’s Modified Eagle Medium (DMEM). Both media were supplemented with 10% fetal bovine serum (FBS), 100 U/mL penicillin–streptomycin antibiotic solution, and 2 mM L-glutamine. The cell cultures were incubated in a humidified incubator at 37 °C with 5% CO_2_. All media and supplements were sourced from Gibco (Waltham, MA, USA).

### 4.9. Cytotoxicity Assay

Cells at a density of 5 × 10^3^ cells/well were seeded onto a 96-well plate. After overnight incubation to allow for cell attachment, the cells were treated with various concentrations of AE for 48 h. Following treatment, 100 µL of the MTT solution (0.5 mg/mL) was added to each well and incubated for another 4 h. The formazan crystals formed were then solubilized using dimethylsulfoxide, and the optical intensity was measured at a wavelength of 570 nm using a microplate reader (VICTOR3/Wallac 1420, Perkin Elmer, Waltham, MA, USA). The percentage of viable cells was calculated as a percentage relative to the control cells. The inhibitory concentration at 50% (IC_50_) was determined using Prism 10 version 10.2.3 (GraphPad Software, Boston, CA, USA).

### 4.10. Apoptosis Assay

For the apoptosis evaluation by annexin-V/PI staining, apoptotic cells were assessed using an apoptosis detection kit (Invitrogen, Waltham, MA, USA). Cells were treated with various concentrations (0–20 μM) of AE for 48 h, washed with cold phosphate-buffered saline, and resuspended in a binding buffer. The cells were then incubated with annexin-V-FITC/PI solution for 15 min at room temperature. The fluorescence intensity of each cell was then analyzed using an EPICS-XL flow cytometer (Beckman Coulter, Indianapolis, IN, USA).

### 4.11. Immunoblot Analysis

Cells were lysed with a lysis buffer composed of 20 mM Tris-HCl (pH 7.5), 1 mM MgCl_2_, 150 mM NaCl, 20 mM NaF, 0.5% sodium metavanadate, 1% nonidet-P40, 0.1 mM phenylmethylsulfonyl fluoride, and protease inhibitor cocktail. Lysis was carried out for 45 min at 4 °C. Following lysis, protein concentrations were measured using a BCA Protein Assay Reagent Kit (Thermo Fisher Scientific, Waltham, MA, USA). The lysates were subsequently separated by sodium dodecyl sulfate-polyacrylamide gel electrophoresis (SDS-PAGE) and transferred to polyvinylidene difluoride (PVDF) membranes (Bio-Rad Laboratories, Hercules, CA, USA). The membranes were blocked with 5% skim milk in Tris-buffer saline containing 0.075% Tween-20 and incubated with specific primary antibodies overnight at 4 °C, followed by incubation with corresponding secondary antibodies at room temperature for 2 h. Rabbit HSP90AA1 (1:1000, CST#8165), mouse anti-GAPDH (1:1000, CST# 97166), HRP-conjugated anti-rabbit (1:1000, CST#7074), and HRP-conjugated anti-mouse (1:1000, CST#7076) were the antibodies used. Protein expression was visualized using an enhanced chemiluminescence system with Immobilon Western chemiluminescent HRP substrate (Merck Millipore, Burlington, MA, USA). Densitometry analysis was performed using Image J software (https://imagej.net/ij/).

### 4.12. Statistical Analysis

Data are presented as the mean ± standard deviation, derived from three independent experiments. Statistical analysis was conducted using Prism 10 version 10.2.3 (GraphPad Software, Boston, CA, USA). One-way analysis of variance (ANOVA) followed by Tukey’s multiple comparison test were employed to evaluate statistical significance, with *p*-value < 0.05.

## 5. Conclusions

This study highlights the potential of AE as a promising candidate for anticancer drug development, particularly in lung cancer. AE significantly induced lung cancer cell death via apoptosis mechanisms. Through network pharmacology and in silico molecular docking and dynamic analyses, HSP90AA1 was identified as a potential molecular target of AE. Subsequent in vitro experiments confirmed that AE induces apoptosis via an HSP90AA1-dependent mechanism. These findings provide valuable scientific insights into the potential of AE for further anticancer drug research and development targeting lung cancer.

## Figures and Tables

**Table 1:** Pathways of adunctin E’s targets in non-small cell lung cancer by the Reactome pathway analysis.

Pathways	Targets
Signal transduction	HDAC7, DD4, MAPK1, HDAC5, CTSD, MMP7, ITGAV, CCNE1, PIK3CA, CDK2, PAK1, NOS3, PLK1, HDAC3, ADORA2B, F2, PAK2, GSK3B, PDE2A, HSP90AA1, PDE5A, RET, RAC1, DRD2, HSP90AB1, PIK3CD, CDK1, MAPK8, RXRA, HDAC2, NTRK1, JAK3, CCKBR, ITGB3, ADORA2A, JAK1
Signaling by receptor tyrosine kinases	MAPK1, CTSD, ITGAV, PIK3CA, PAK1, NOS3, HDAC3, PAK2, HSP90AA1, RAC1, HDAC2, NTRK1, JAK3, ITGB3, ADORA2A
Degradation of the extracellular matrix	CTSD, MMP7, MMP13, CAPN2, PRSS1, MMP1, CTSB, CAPN1, CAPNS1
TP53 regulates transcription of cell cycle genes	AURKA, CCNB1, CCNE1, CDK2, CDK1, CCNE2
MAPK family signaling cascades	MAPK1, PIK3CA, PAK1, PAK2, RET, RAC1, CDK1, JAK3, ITGB3, JAK1
Apoptosis	HSP90AA1, MAPK1, PAK2, TLR4, MAPK8, DAPK1, DAPK3, DAPK2
Cell cycle	MAPK1, AURKA, CCNB1, CCNE1, CDK2, PLK1, GSK3B, HSP90AA1, HSP90AB1, CDK1, CCNE2
PI3K/AKT signaling in cancer	PIK3CA, GSK3B, RAC1, PIK3CD, NTRK1

**Table 2:** Interaction strength between 22-(40py)-JA adunctin E and potential targets.

Targets	PDB	Binding Energy (kcal/mol)	Ligand Efficiency (kcal/mol per Heavy Atom)	Number of Interactions			
				Hydrogen	van der Waals	Hydrophobic	Electrostatic
HSP90AA1	4BQG	−10.1	0.32	-	12	9	-
MAPK1 (ERK2)	1WZY	−7.7	0.24	1	11	11	-
PIK3CA	6DGT	−8.1	0.25	2	7	3	1

## Data Availability

All data supporting the findings of this study as well as Supplementary Materials are available within the paper and published online.

## Supplementary Materials

The following supporting information can be downloaded at: https://www.mdpi.com/article/10.3390/ijms252111368/s1.
