# Supplementary material for: Adunctin E from Conamomum rubidum Induces Apoptosis in Lung Cancer via HSP90AA1 Modulation: A Network Pharmacology and In Vitro Study
Source: Int J Mol Sci. 2024 Oct 22;25(21):11368. doi: 10.3390/ijms252111368 (PMC11546842; doi:10.3390/ijms252111368)
Supplement: Supplementary file 1 [file ijms-25-11368-s001.zip › ijms-3239007-supplementary.pdf]

Supplementary information for

Adunctin E from *Conamomum rubidum* Induces Apoptosis in Lung Cancer via HSP90AA1 Modulation: A Network Pharmacology and *In Vitro* Study

Iksen Iksen[1], Natsaranyatron Singharajkomron[1], Hien Minh Nguyen[2], Hanh Nhu Thi Hoang[3,4], Duc Viet Ho[4], Varisa Pongrakhananon[1,5*]

[1] Department of Pharmacology and Physiology, Faculty of Pharmaceutical Sciences, Chulalongkorn University, Bangkok, Thailand; ikseniksen08@gmail.com (I.I.); Natsaranyatron.S@gmail.com (N.S.); varisa.p@pharm.chula.ac.th (V.P.)
[2] Faculty of Pharmacy, Ton Duc Thang University, Ho Chi Minh City, Vietnam; nguyenminhhien@tdtu.edu.vn (H.M.N)
[3] Faculty of Engineering and Food Technology, Hue University of Agriculture and Forestry, Hue University, Hue city, Vietnam; htnhanh@hueuni.edu.vn (H.N.T.H)
[4] Faculty of Pharmacy, Hue University of Medicine and Pharmacy, Hue University, Hue city, Vietnam; hvietduc@hueuni.edu.vn (D.V.H)
[5] Preclinical Toxicity and Efficacy Assessment of Medicines and Chemicals Research Unit, Chulalongkorn University, Bangkok, Thailand
* Correspondence: varisa.p@pharm.chula.ac.th

Table S1. Pharmacokinetic parameters of adunctin E was analyzed by pkCSM.

Property	Model Name	Predicted Value	Unit
Absorption	Water solubility	-6.533	Numeric (log mol/L)
	Caco2 permeability	0.5	Numeric (log Papp in 10^{-6} cm/s)
	Intestinal absorption (human)	92.462	Numeric (% Absorbed)
	Skin Permeability	-2.855	Numeric (log Kp)
	P-glycoprotein substrate	Yes	Categorical (Yes/No)
	P-glycoprotein I inhibitor	Yes	Categorical (Yes/No)
	P-glycoprotein II inhibitor	Yes	Categorical (Yes/No)
Distribution	VDss (human)	-0.249	Numeric (log L/kg)
	Fraction unbound (human)	0	Numeric (Fu)
	BBB permeability	-0.582	Numeric (log BB)
	CNS permeability	-2.675	Numeric (log PS)
Metabolism	CYP2D6 substrate	No	Categorical (Yes/No)
	CYP3A4 substrate	Yes	Categorical (Yes/No)
	CYP1A2 inhibitor	No	Categorical (Yes/No)
	CYP2C19 inhibitor	Yes	Categorical (Yes/No)

	CYP2C9 inhibitor	Yes	Categorical (Yes/No)
	CYP2D6 inhibitor	No	Categorical (Yes/No)
	CYP3A4 inhibitor	Yes	Categorical (Yes/No)
Excretion	Total Clearance	0.691	Numeric (log ml/min/kg)
	Renal OCT2 substrate	No	Categorical (Yes/No)
Toxicity	AMES toxicity	No	Categorical (Yes/No)
	Max. tolerated dose (human)	0.021	Numeric (log mg/kg/day)
	hERG I inhibitor	No	Categorical (Yes/No)
	hERG II inhibitor	No	Categorical (Yes/No)
	Oral Rat Acute Toxicity (LD50)	2.77	Numeric (mol/kg)
	Oral Rat Chronic Toxicity (LOAEL)	1.472	Numeric (log mg/kg_bw/day)
	Hepatotoxicity	No	Categorical (Yes/No)
	Skin Sensitisation	No	Categorical (Yes/No)
	T.Pyriformis toxicity	0.322	Numeric (log ug/L)
	Minnow toxicity	-2.001	Numeric (log mM)

Table S2. Targets of adunctin E were evaluated by Swiss Target Prediction database and Similarity Ensemble Approach (SEA).

ACACB	DAPK2	ITGB3	NDUFA11	NDUFB7	PAK2	SLC28A3
ADORA2A	DAPK3	JAK1	NDUFA12	NDUFB8	PDE10A	ST3GAL1
ADORA2B	DRD2	JAK3	NDUFA13	NDUFB9	PDE11A	ST6GAL1
APH1A	DRD3	KLK1	NDUFA2	NDUFC1	PDE2A	STK17A
APH1B	DRD4	KLK2	NDUFA3	NDUFC2	PDE4A	STK17B
AURKA	EHMT2	KLKB1	NDUFA4	NDUFS1	PDE4B	TLR4
BDKRB1	F10	MAPK1	NDUFA4L2	NDUFS2	PDE5A	TNNC1
CA4	F2	MAPK8	NDUFA5	NDUFS3	PDF	TNNI3
CALCRL	FAP	MELK	NDUFA6	NDUFS4	PIK3CA	TNNT2
CAPN1	FUT5	MMP1	NDUFA7	NDUFS5	PIK3CD	TRPV1
CAPN2	GABRA1	MMP13	NDUFA8	NDUFS6	PIM1	
CCKAR	GABRA2	MMP7	NDUFA9	NDUFS7	PIM2	
CCKBR	GABRA3	MMP8	NDUFAB1	NDUFS8	PITRM1	
CCNB1	GCK	MPO	NDUFAF1	NDUFV1	PLK1	
CCNE1	GSK3B	MT-ND1	NDUFAF2	NDUFV2	PREP	
CCNE2	HCRTR1	MT-ND2	NDUFAF3	NDUFV3	PRKCQ	
CDK1	HDAC10	MT-ND3	NDUFAF4	NISCH	PRSS1	
CDK2	HDAC2	MT-ND4	NDUFB1	NOS1	PSEN1	
CHRM4	HDAC3	MT-ND4L	NDUFB10	NOS2	PSEN2	
CHRNA7	HDAC5	MT-ND5	NDUFB11	NOS3	PSENEN	
CTSB	HDAC7	MT-ND6	NDUFB2	NPY5R	PTGS2	
CTSD	HSD17B2	NAAA	NDUFB3	NR3C2	RAC1	

CTSS	HSP90AA1	NCSTN	NDUFB4	NTRK1	RET	
CYP19A1	HSP90AB1	NDUFA1	NDUFB5	P2RX7	RORC	
DAPK1	ITGAV	NDUFA10	NDUFB6	PAK1	RXRA	

Table S3. Common targets of adunctin E and non-small cell lung cancer.

ADORA2A	CDK2	FAP	JAK3	NOS1	PLK1
ADORA2B	CHRNA7	GABRA3	MAPK1	NOS2	PRSS1
AURKA	CTSB	GSK3B	MAPK8	NOS3	PTGS2
CA4	CTSD	HDAC2	MELK	NTRK1	RAC1
CAPN1	CYP19A1	HDAC3	MMP1	P2RX7	RET
CAPN2	DAPK1	HDAC5	MMP13	PAK1	RORC
CAPNS1	DAPK2	HDAC7	MMP7	PAK2	RXRA
CCKBR	DAPK3	HSP90AA1	MPO	PDE2A	ST6GAL1
CCNB1	DRD2	HSP90AB1	NDUFA13	PDE5A	STK17A
CCNE1	DRD4	ITGAV	NDUFA4L2	PIK3CA	TLR4
CCNE2	EHMT2	ITGB3	NDUFB10	PIK3CD	TRPV1
CDK1	F2	JAK1	NDUFS2	PIM1	

Table S4. The top 16 core targets of adunctin E against non-small cell lung cancer were ranked according to the degree values.

Name	Degree	Betweenness Centrality	Closeness Centrality	Clustering Coefficient
Heat Shock Protein 90 Alpha Family Class A Member 1 (HSP90AA1)	12	0.311205918	0.493670886	0.27272727
Mitogen-Activated Protein Kinase 1 (MAPK1)	12	0.308104499	0.475609756	0.15151515
Cyclin Dependent Kinase 2 (CDK2)	9	0.0513161	0.375	0.58333333
Cyclin Dependent Kinase 1 (CDK1)	9	0.068958729	0.375	0.55555556
Phosphatidylinositol-4,5-Bisphosphate 3-Kinase Catalytic Subunit Alpha (PIK3CA)	9	0.359777844	0.475609756	0.22222222
Heat Shock Protein 90 Alpha Family Class B Member 1 (HSP90AB1)	8	0.062993095	0.414893617	0.42857143
Toll Like Receptor 4 (TLR4)	8	0.168755109	0.433333333	0.17857143
Aurora Kinase A (AURKA)	7	0.008092747	0.30952381	0.61904762
Cyclin B1 (CCNB1)	7	0.002519118	0.288888889	0.71428571
Polo Like Kinase 1 (PLK1)	7	0.039691011	0.367924528	0.66666667
Glycogen Synthase Kinase 3 Beta (GSK3B)	6	0.060286385	0.393939394	0.46666667
Cyclin E2 (CCNE2)	5	2.70E-04	0.284671533	0.9
Cyclin E1 (CCNE1)	5	0.003518082	0.3046875	0.7
Histone Deacetylase 2 (HDAC2)	5	0.55	1	0.3
Nitric Oxide Synthase 2 (NOS2)	5	0.019828148	0.375	0.4
Phosphatidylinositol-4,5-Bisphosphate 3-Kinase Catalytic Subunit Delta (PIK3CD)	5	0.033558255	0.371428571	0.5

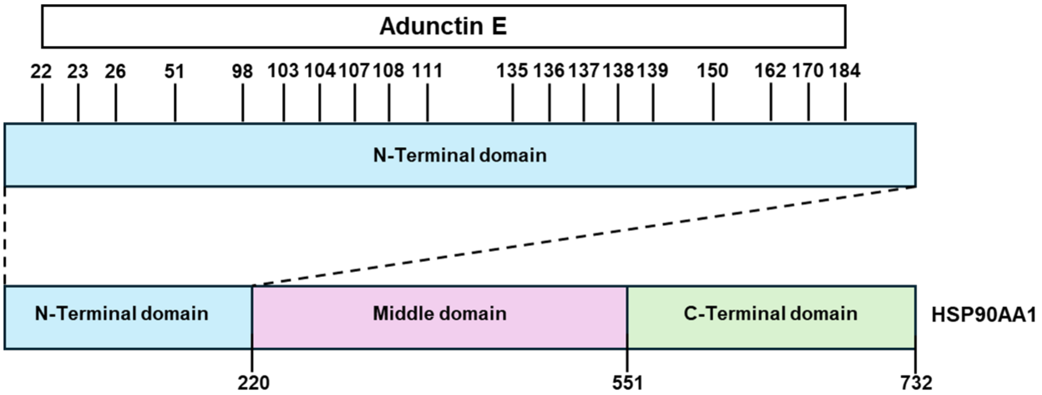

Figure S1. Adunctin E interacts with multiple residues on the N-terminal domain of HSP90AA1, with a significant binding observed at THR184, a key residue involved in ATP binding and hydrolysis.

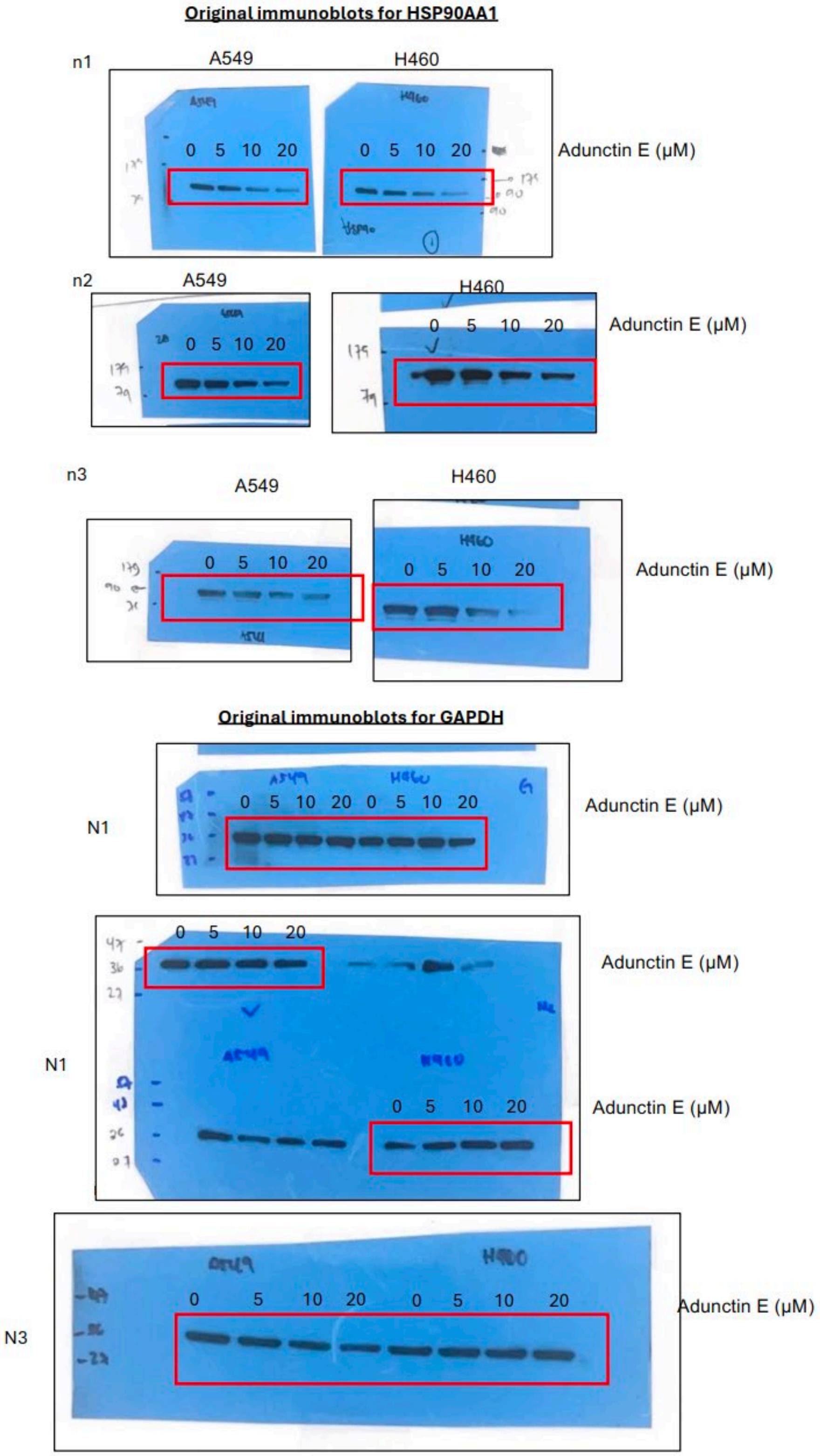

Figure S2. The original blots for Figure 8A.
